# A Neuromuscular Interface for Robotic Devices Control

**DOI:** 10.1155/2018/8948145

**Published:** 2018-07-22

**Authors:** Innokentiy Kastalskiy, Vasily Mironov, Sergey Lobov, Nadia Krilova, Alexey Pimashkin, Victor Kazantsev

**Affiliations:** Center for Translational Technologies, Nizhny Novgorod Neuroscience Center, National Research Lobachevsky State University of Nizhny Novgorod, Gagarin Ave. 23, Nizhny Novgorod 603950, Russia

## Abstract

A neuromuscular interface (NI) that can be employed to operate external robotic devices (RD), including commercial ones, was proposed. Multichannel electromyographic (EMG) signal is used in the control loop. Control signal can also be supplemented with electroencephalography (EEG), limb kinematics, or other modalities. The multiple electrode approach takes advantage of the massive resources of the human brain for solving nontrivial tasks, such as movement coordination. Multilayer artificial neural network was used for feature classification and further to provide command and/or proportional control of three robotic devices. The possibility of using biofeedback can compensate for control errors and implement a fundamentally important feature that has previously limited the development of intelligent exoskeletons, prostheses, and other medical devices. The control system can be integrated with wearable electronics. Examples of technical devices under control of the neuromuscular interface (NI) are presented.

## 1. Introduction

Development of neurointerface technology is a topical scientific focus, with the demand for such systems driven by the need for humans to communicate with numerous electronic computing and robotic devices (RD), for example, in medical applications such as prosthetic limbs and exoskeletons. At present, multichannel recording of neuromuscular activity and the development of neurointerface applications that implement unique mechanisms for high-dimensional data processing are areas of major interest.

One of the most suitable signals aiming at controlling external RDs is electromyographic (EMG) activity. Multichannel signals from the human peripheral nervous system have been previously successfully used to control external devices and novel methods of EMG acquisition and control strategies have recently been implemented [1–8]. When controlling anthropomorphic RD, the human pilot independently coordinates and plans the trajectory of motion using the massive computing power of the human brain [9, 10]. The use of afferent neural pathways allows the activation of biological feedback; using this principle is fundamentally important to the development of rehabilitation exoskeletons, prostheses, and various other medical applications.

The disadvantages of using EMG interfaces in rehabilitation are the presence of muscle fatigue and insufficient residual muscle activity. On the other hand electroencephalographic (EEG) interfaces proved to be the best due to a direct link to the nervous system by measurement of brain activity during therapy [11, 12]. The brain mechanisms that enable humans to facilitate the control of external devices remain largely unknown. However, despite this knowledge gap, appropriate collection, detection, and classification can enable brain-controlled signals from the human body to be utilized for highly efficient and even intelligent control of multiparameter RDs. But brain-machine interfaces (BMI) have some limitations such as low reliability and accuracy when it comes to complex functional task training.

A possible solution to these problems is the combined use of the advantages of both types of interfaces. Such interfaces are called hybrid, for example, hybrid BMI (hBMI); the use of EMG input here can lead to a more accurate classification of EEG patterns [13–15]. However, the task of developing an EMG interface is still relevant.

Considering the problem of motion recognition and decoding of EMG signals, note that there are several generally applicable methods of software signal processing: linear discriminant analysis (LDA) [20], support vector machines (SVM) [21], artificial neural networks (ANN) [22], fuzzy algorithms [22, 23], etc.

Despite significant progress in the field of machine learning and its application in medical tasks [24], algorithms are still based on applying ANN technologies and solving optimization problems. Creation of a universal algorithm that can adapt to different conditions in a technical control system was proven theoretically impossible, at least in the context of existing theories [25, 26]. Compared to traditionally controlled electronic devices, neurocontrolled devices may offer the advantage of adapting due to human brain plasticity.

The present study focuses on the development of methods and technologies for remote control of RDs in specific applications. The objective was to integrate human bioelectrical signals into a control loop. Online collection and interpretation of multisite EMG signals were performed to control a variety of robotic systems. Technical solutions were developed to associate patterns of muscular activity (and human brain, if possible) with the commands to the controlled object by employing a user-defined translation algorithm. EMG interface solution is driven by multilayer ANN feature classifier. User-defined programmable function translates sensory signals into motor commands to successfully control a variety of existing commercial RDs.

## 2. Methods

### 2.1. EMG Array

Multielectrode array (EMG array) was designed as a data acquisition system that detects the EMG signals associated with wrist gestures. Monitoring of the signals from several muscles was performed simultaneously (for example, the muscles of the forearm, involved in making gestures:* m. brachioradialis*,* m. flexor carpi radialis*,* m. palmaris longus*,* m. flexor carpi ulnaris*, etc.). First layout contained six pairs of standard medical Ag/AgCl electrodes, which are often used for surface EMG recording. The electrodes were placed on the flexible fabric, which was put on the forearm at a distance of about 1/3 from the elbow to the wrist. The EMG array was suitable for several hours of recording.

**Figure 1:**
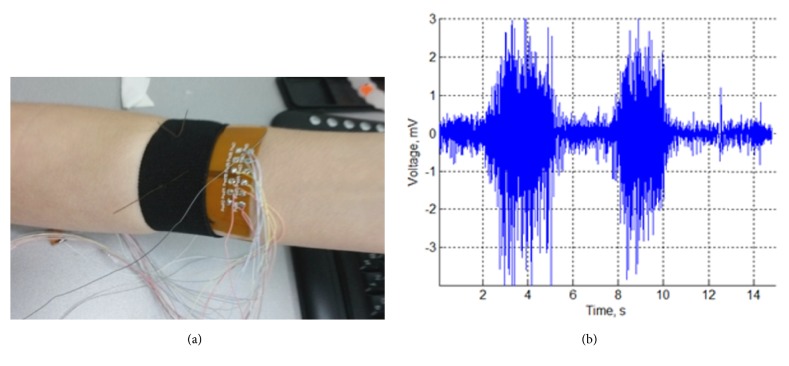
Multielectrode array for EMG signal recording. (a) Medical Ag/AgCl electrodes of the flexible EMG array used to record the muscles activity. (b) EMG signal from one electrode of the array. Signal contains two periods of muscle contraction.

An array was developed using commercial technology of printing circuit board (PCB) flexible electronics: flexible substrate made of polyimide with six pairs of silver-coated (99.9% silver) planar electrodes (Figure 1(a)). Registration was performed in bipolar mode; i.e., the muscle signal was obtained by pairs of electrodes. The reference electrode was mounted close to the elbow. An example of EMG signal on one electrode is shown in Figure 1(b).

### 2.2. Multichannel Signal Registration and Classification Using an Artificial Neural Network

Ten healthy volunteers aged 20 to 42 years were recruited for experimental purpose. All persons had different physique (asthenic: 1, hypersthenic: 2, and normosthenic: 7) and had no previous experience in dealing with EMG interfaces. Two series of nine gestures each were performed in a random order.

Next, registered signals for nine static hand gestures, such as motor patterns, were classified. The first series was the learning set; the second series was the testing set. The data flow (EMG amplitudes) **x**(*t*) ∈ *ℝ*^6^ was divided into 200 ms overlapping time windows at a 100 ms step (*t* = 0,1,2,… is the discrete time with the sampling rate of 1 kHz). Then the moving root mean square (RMS) values of the EMG signal along each channel independently over time were calculated in order to extract the features of the multichannel signal.

(1) $$\begin{array}{c} RMS\left(\;t\;\right)=\sqrt{\frac{1}{N}\overset{N-1}{\underset{n=0}{\sum }}x{\left(t-n\right)}^{2}}, \end{array}$$

where* N* = 200 is the number of samples in a time window and* t* =* Mk* (*k* = 2,3,4,…) with* M* = 100 being the time shift between consecutive windows. Each 50 ms RMS was fed to a multilayer artificial neural network (ANN) for feature classification.

The network neurons apply weighted sum over inputs,* z*_*i*_, and use sigmoidal activation function (2) to generate output, **y**:

(2) $$\begin{array}{c} y=\frac{1}{1+e^{-\sum_{i}w_{ij}z_{i}}}, \end{array}$$

where *w*_*ij*_ are the synaptic weights of neuron* j*. The learning, i.e., adjustment of the neuron weights **w**, is achieved by the backpropagation algorithm [27]. During the learning, the weight *w*_*ij*_ is corrected proportionally to the error *δ*_*j*_ introduced by the neuron* j *when the current sample is fed to the network input:

(3) $$\begin{array}{c} {\Delta w}_{ij}=\eta \delta_{j}x_{i}, \end{array}$$

where *η* is learning rate and* x*_*i*_ is the signal from neuron* i* to neuron* j*. Running through the network of all samples makes up an epoch. As a rule, a large number of epochs are required for training. Each basic gesture corresponds to a single target class. Thus, each neuron of the last layer should produce “1” for one class and “0” for the others.

The classification error was calculated for the training and testing sets as the rate of incorrectly recognized samples. It served as a criterion to stop the learning procedure as soon as the error started increasing on test samples. On average the learning process required about 5000 training epochs and took less than 1 min on a standard Intel Core i5 PC.

Once the learning is deemed finished, online controlling of a robotic device can be enabled. To introduce a proportional control an approach similar to that described in [8] was employed. The muscle effort is evaluated by the mean absolute value (MAV) averaged over all EMG sensors:

(4) $$\begin{array}{c} MAV\left(\;t\;\right)=\frac{1}{NK}\overset{K}{\underset{k=1}{\sum }}\;\overset{N-1}{\underset{n=0}{\sum }}\left|\;x_{k}\left(\;t-n\;\right)\;\right|, \end{array}$$

where* K* is the number of EMG channels (in our case* K* = 6). Then the actuator's rotation speed is set proportional to the MAV.

### 2.3. Software and Tested Robotic Devices

The RDs tested in this study were the LEGO NXT Mindstorms mobile robot (LEGO, Denmark) [7, 8], the NAO humanoid robot (Aldebaran, France), and an exoskeleton “Ilya Muromets” (UNN, Russia) [28]. The standard software development kits (SDKs) of each device were used. The connections between the control device and tested RDs were wireless: Bluetooth for LEGO or Wi-Fi for NAO and the exoskeleton. If the SDK had a support of movement instructions, a direct macrocommand was sent (e.g., “go forward” for the NAO and exoskeleton). Otherwise, the required macrocommands were implemented by the software and sent to the elementary command of the device (e.g., “rotate motor A with speed x%” for LEGO).

**Figure 2:**
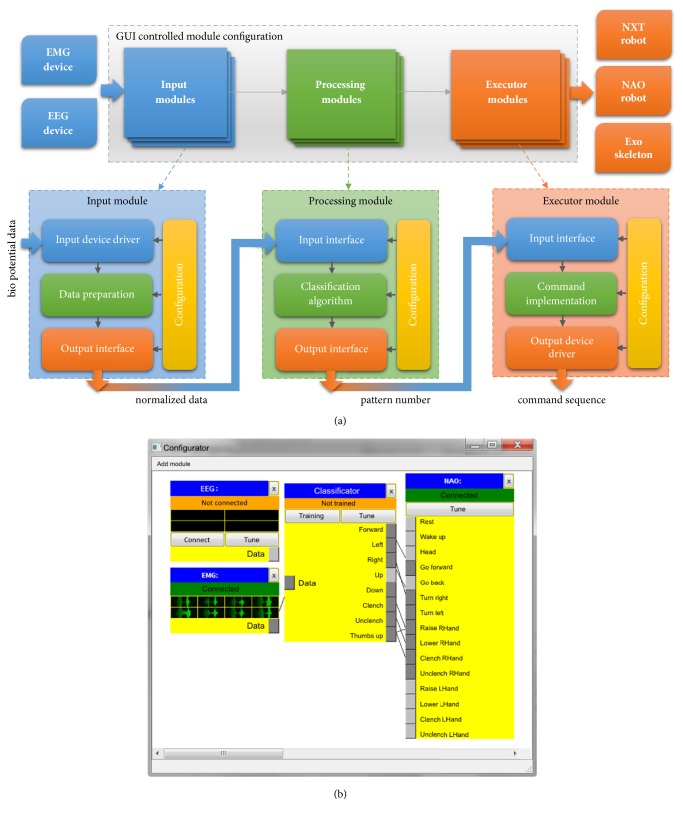
The “Configurator” for the programmable translator of NI. (a) Flow chart. (b) Main window of the software module. It allows for setting the modalities for processing and the type of translation of the input signal of the human pilot to the output one on device actuators.

To configure the parameters of the signal translator, a special software module was developed (Figure 2). The software contains GUI interface that allows creating a test bed configuration. Various modules can be added and a different modules relationship configuration can be set up. Also, the operator has the ability to change the specific settings for each module.

Three types of modules are used: input modules, processing modules, and executor modules. Each output of any input module can be connected to one or more free input slots of any processing module, and likewise each output of any processing module can be connected to one or more free input slots of any executor module.

The input modules provide an interface with data acquisition devices, such as EMG and EEG adapters. One of the tasks of input modules is preprocessing (filtering, resampling) of incoming data and their normalization. The normalized data is then transferred to the processing unit with which this input module was connected in the “Configurator” (Figure 2(b)).

The processing modules perform the classification tasks based on the selected algorithm. The result of the classifier operation is the number of the recognized pattern, which is transmitted to the corresponding module of the executive device.

The executive device module is a driver that communicates with the executive device and converts the pattern number received from the processing module into a command sequence of a particular device to perform the desired action.

## 3. Results

### 3.1. EMG Data Acquisition

The parameters of the EMG signal recorded using NI were comparable to similar systems described in the literature [4, 16, 19, 29–31]. The design of the electrode array enabled stable signal recording and could potentially be used to further develop neurointerfaces for prosthetic limb control in medical and rehabilitation applications or commercial interfaces for everyday use.

One of the most important characteristics of the hardware amplifier of NI is the low noise of the raw signal. In the case of the input signal with approximate amplitude of 100 mV a mean signal-to-noise-ratio (SNR) was 11.9 ± 0.5 dB, for 200 mV – 19.3 ± 0.7 dB, and for 500 mV – 29.2 ± 0.9 dB. Series of SNR values measured experimentally showed stability of this characteristic but in the majority of cases such a level cannot be considered very high. However, normal values in these signal amplifiers (usually 50 dB and more) are indicated for measurements performed in ideal conditions.

### 3.2. ANN Parameters Optimization

To optimize ANN performance, gesture recognition on the same datasets of EMG signals (patterns of the RMS signals) was performed. The number of layers in the ANN, the number of neurons in hidden layers, and the learning rate were varied. The ANN error dropped significantly between one and two layers and then slightly increased as the number of layers increased up to eight, while learning time increased significantly. A similar increase in ANN error was obtained as the number of neurons in the hidden layers increased from 8 to 16. Thus, a network with two layers and eight neurons in the hidden layer was selected for further experiments. It was also found that a learning rate of 0.01 led to optimal learning error and learning time. This is learning rate dimensionless parameter of standard backpropagation algorithm. Thus, this learning rate was used in all experimental tests of the interface.

### 3.3. Neuromuscular Interface Performance

The software and hardware system implement both command control based on pattern classification and proportional control based on muscle effort estimation. Several schemes for combining these strategies were previously suggested [7, 32]. In particular, the patterns for controlling direction of movement and muscle effort to control speed were recognized.

Note that personal classification accuracy varied significantly [33]. For example, the accuracy of recognition for nine patterns for ten users ranged from 86.5 to 98.5%. In this regard, the possibility of improving personal performance by training the user was explored.

To measure the personal progress an index of neurointerface performance (NP) was introduced:

(5) $$\begin{array}{c} NP=\ln\left(\;\frac{E_{i}}{E_{1}}\;\right), \end{array}$$

where* E*_*i*_ is the error of EMG pattern classification on the current training day and* E*_1_ is the error on the first day. Note that on the first day* NP* is equal to 0 always. A positive* NP* value means degradation of the interface performance, and a negative value means an improvement.

**Figure 3:**
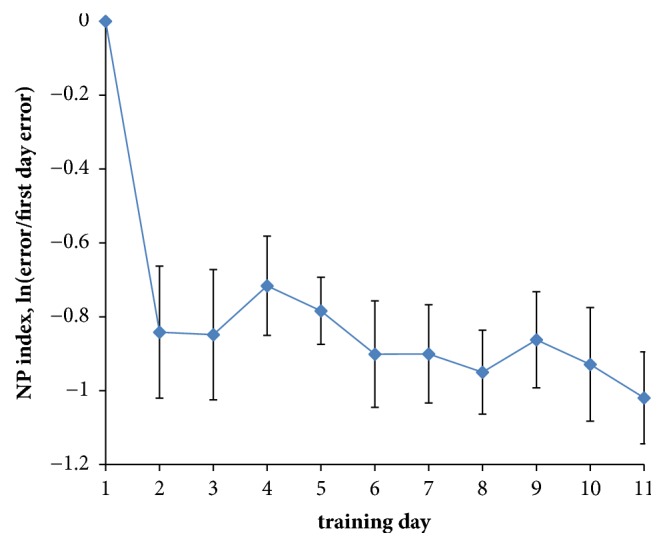
Evolution of neurointerface performance (NP index) during training. Averaged data for 10 users are shown. Error bars correspond to standard deviations.

Eight of the ten subjects showed a positive improvement in performance after several days of training including playing a training game with the EMG interface.  Figure 3 illustrates the improvement in terms of* NP* index. The majority of progress was achieved on the second day of training. This is acceptable, given that a short training course would be necessary before any user could effectively operate an EMG interface.

Our previous study reported the accuracy of the pattern classification algorithm used in this NI was 92% ± 4% for the nine gestures and 97% ± 2% for six gestures in the command control mode [32]. This high accuracy rate is very close to the attainable limit (“error-free”) in the development of human-machine interfaces.

A detailed comparison of the characteristics of NI developed in this study and other devices is shown in Table 1.

Neuromuscular interface consisted of an EMG module that permitted control of external RDs, including existing commercial ones, using muscle effort patterns. In the future, our device could also be improved by adding an EEG module that permits control of RDs using both brain intention and EMG patterns.

Overall, the hardware and software system described in this study could successfully interpret the bioelectric activity signals from the pilot into robotic commands to achieve correct control of the tested RDs.

## 4. Discussion

Trying to develop an ideal human-machine interface, one must keep in mind and improve not only its technical component. It requires understanding how much a person can limit system performance. Despite a relatively high mean fidelity, neurointerfaces still exhibit strong variance in the accuracy of gesture recognition among different users. Our recent study showed that the factors determining the performance of neurointerfaces were the degree of muscle cooperation and the amount of the body fatty tissue. A person can improve his/her performance in the long run by doing sports or fitness (nonspecific training) or even in a short period of time training with NI (specific training) [33]. It is crucial to identify “problematic” gestures.

In this study, users were informed of their errors in execution of gestures, and as a result, on the second day of testing, their performance improved. Outside the study remains an important question about the motivation. In our experiments, a significant drop in motivation was observed already on the second week. Most likely, people who really need a NI (for example, amputees) will be able to show a more impressive dynamics of training.

One of the drawbacks of the proposed NI hardware implementation is the wire communication channel of the EMG array. It is not suitable for long-term unconstrained use. However, it can be overcome by developing a portable amplifier with a wireless transmitter driven by Bluetooth 4.0 protocol. The latter has high noise immunity and low requirements to the electromagnetic environment. In this embodiment, the sensors will be quite more cumbersome, but there will be no hand obstructions or electrode wires imposing constraints on allowable movements.

Nevertheless, the use of high-density surface electromyography (HDEMG) [3] can bring the approach greater solidity. It can add redundancy and is more immune from movement artifacts (electrode slippage, etc.), with the potential to significantly improve decoding reliability.

On the other hand the disadvantage of a HDEMG is its high power consumption. But advanced algorithms of active channel selection can lead to low power consumption per channel, which enables operation for long periods of time on miniature batteries.

In the future the NI can be conveniently embedded into wearable garments and worn unobtrusively by the operator. No extra setup time is required for placement of individual electrodes, fine alignment, etc.

The functioning of a device combining EMG and EEG modalities imposes certain difficulties in implementing the control strategy. Such an implementation is seen as promising in the case of rehabilitation of severe motor impairment. EEG should be used as a trigger to confirm of a movement execution. The output of the gesture recognition can be mapped into various command libraries for different control modes.

Being designed for either medical rehabilitation or general consumer, the NI must have characteristics that take into account the fundamental computational aspects of the brain. Employment of brain information processing power in control applications still has many questions debated. On the one hand, possibilities of modern electronics together with advanced ANN classification algorithms permit achieving quite fast rather precise multiparameter human-machine interfaces as has been demonstrated in the present study. On the other hand, the NI power is still limited by ultimate need of human concentration to implement the proportional control. The development of advance training algorithms and tools to monitor pilot's concentration during control gives challenges for further work in this direction. Another, more fundamental question is how many parameters and external devices one pilot can navigate simultaneously? Theoretically, the number of muscles of the body simultaneously controlled by the brain is huge. For example, a simple grasping finger movement involves up to 50 muscles [34]. They represent muscle synergies that consisted of groups of muscles worked in a coherence to implement a given motor task.

In this context in nearest future, feasibly, properly configured multisite EMG human-machine interface will be able to provide adaptive control in real time of many parameters/limbs/actuators including ones with remote control. In other words, nervous system (e.g., the peripheral one) will be integrated with machine controllers and interpreted by brain as “natural” extension of the body. To work like that different feedback channels in addition to purely biological feedback (e.g., visual, olfactory) might be needed to develop.

Further research in this direction not only has obvious applied perspective in rehabilitation medicine and industrial robotics but also will shed light on fundamental principles of motor control implemented by our brain.

## 5. Conclusions

A technical solution for collecting, decoding, and translating multichannel biometric data to control a variety of external RDs was described. Novel algorithms for the classification of human bioelectric activity patterns were developed. In particular, the approach to implement muscle activity patterns classification using artificial neural network was proposed. It permitted classifying up to nine patterns with very high average accuracy (98.5% for some persons) relative to other systems.

Experimental tests of developed recording and decoding system were performed. During operational testing, NI functioned correctly when controlling existing commercial RDs such as the Aldebaran Robotics NAO and an exoskeleton for the lower limbs.

## Figures and Tables

**Table 1:** Comparison of various myoelectric control devices.

Indicator measured	NI	Fougner et al., 2012, [16]	Wurth et al., 2014, [17]	Jiang et al., 2012, [18]	Hahne et al., 2014, [19]	Hahne et al., 2016, [4]	Earley et al., 2016, [6]
Average recognition accuracy	92.5%	-	96%	>90%	-	~90%	-

Control	Command and proportional	Consistent proportional	Motion pattern recognition. Proportional	Proportional	Proportional	Command and proportional	Motion pattern recognition. Proportional

Classifier	ANN (perceptron)	LDA	LDA	ANN (perceptron)	ANN (perceptron)	Linear regression	LDA

Number of gestures / degrees of freedom (DoF)	9 gestures	5 gestures	2 DoF, 5 gestures	3 DoF	2 DoF, 4 gestures	2 DoF, 4 gestures	8 gestures

Number of EMG channels /sensors	8 for recording + 1 reference	5	6	7 pairs for each forearm	192-channel electrode array in the monopolar configuration	4 for each type of electrode	12 pairs of bipolar electrodes
